# Understanding the contextual and cultural influences on women’s modern contraceptive use in East Uganda: A qualitative study

**DOI:** 10.1371/journal.pgph.0000545

**Published:** 2022-08-15

**Authors:** Amrita Namasivayam, Philip J. Schluter, Sarah Namutamba, Sarah Lovell

**Affiliations:** 1 School of Health Sciences, University of Canterbury—Te Whare Wānanga o Waitaha, Christchurch, New Zealand; 2 Medical School—General Practice Clinical Unit, The University of Queensland, Brisbane, Australia; 3 Institute of Public Health, Makerere University, Kampala, Uganda; Babcock University, NIGERIA

## Abstract

Unmet need for contraception, defined as the percentage of women who are sexually active and want to avoid, space or limit pregnancies, but are not using a method of contraception, stands at 28.4% of all married women in Uganda. An understanding of women’s contraceptive behaviours, and the motivations that drive these, are key to tackling unmet need, by way of designing, implementing and improving family planning programs to effectively meet the needs of different population groups. This qualitative study sought to understand women’s contraceptive use and identify strategies to strengthen contraceptive uptake among women in the Busoga region of east Uganda (chosen due to its low contraceptive prevalence of 31.3% and high unmet need of 36.5% among married women of reproductive age). Six focus group discussions were conducted with single and married women across different age groups (18–24, 25–34, and ≥ 35 years), living in three urban and three rural districts. Thematic analyses of the data highlighted three major themes pertaining to the complex, multi-level nature of contributors to unmet need and women’s use of contraception in the Busoga region. Within a largely patriarchal society, women had to navigate many obstacles. Some of these included: fears about contraceptive side effects; partner opposition, community beliefs and stigma that dissuaded contraceptive use; traditional gender and socio-cultural norms that dictated women’s fertility choices; and service delivery limitations. Changing community narratives about family planning through testimonies from satisfied users, increasing male acceptance of contraception, and encouraging joint-decision making on matters of reproductive health are strategic focal areas for family planning initiatives to effectively tackle the problem of unmet need among women, and make contraceptives more accessible to women in Uganda.

## 1. Introduction

Uganda has seen substantial reductions in maternal mortality and morbidity, and improvements in access, affordability and availability of reproductive health and family planning service provision in recent decades [1]. Despite these improvements, unmet need for contraception (Unmet need is defined as the percentage of women who are sexually active and want to avoid, space or limit pregnancies, but are not using a method of contraception) in Uganda remains high, at 28.4% of married women of reproductive age, and 31.9% of sexually active, unmarried women [2]. Uganda has one of the highest fertility rates in the east African region, with a total fertility rate of 5.4 children per woman at the national level [2]. With short spacing between births being common, and unmet need for contraception high among postpartum women, it is estimated that 56.0% of all pregnancies and 37.0% of pregnancies among married women in Uganda are unintended [3, 4].

The Ugandan government however, recognizes that family planning is central to economic development. In 2014, Uganda’s Ministry of Health (MOH), in collaboration with the United Nations Population Fund and several implementing partners, developed the Uganda Family Planning- Costed Implementation Plan (FP-CIP), 2015–2020. This was intended to be an overarching policy document to guide national level strategies and programs aimed at increasing and improving family planning initiatives and access, with specific goals to reduce “unmet need for family planning to 10% and increase the modern contraceptive prevalence rate to 50% by 2020” [5]. While FP-CIP 2015–2020 targets and outcomes are being evaluated, a FP-CIP II for 2021–2025 is being formulated at present. In 2021, as part of renewed Family Planning 2030 commitments, Uganda committed to increasing modern contraceptive prevalence rate for all women from 30.4% in 2020 to 39.6% by 2025 and reducing unmet need from 17.0% in 2020 to 15.0% by 2025 [6].

In the context of Uganda, the reasons for unmet need are many. Demographically, women’s higher educational levels and socioeconomic status as well as higher age and parity and urban place of residence (versus rural) show associations with higher rates of contraceptive use [7–10]. Older women and women of higher parity are more likely to use contraception to limit their number of pregnancies, and women’s economic independence and empowerment favour contraceptive use [8, 11–13].

While awareness about contraception and different contraceptive methods is almost universal among women in Uganda, research points to a discrepancy between levels of contraceptive uptake and contraceptive knowledge [14, 15]. This contrast was central in determining the directives and objectives of the FP-CIP 2015–2020 [5], with the Plan seeking to influence social norms and attitudes with a specific focus on addressing the: “myths, misconceptions, and side effects and improve acceptance and continued use of family planning to prevent unintended pregnancies” [5]. This priority reflects a growing body of research that recognises community beliefs and acceptance of contraceptive use can influence women’s own contraceptive knowledge, choices and behaviour [16]. Misconceptions and fears of both real and perceived negative contraceptive side effects are widespread barriers to contraceptive use [17–20]. The reliance on peers and community beliefs as a source of family planning information remains a challenge in addressing myths and fears associated with contraceptive side effects [21]. Social norms are particularly influential when community attitudes towards contraceptive use conflict with women’s own fertility desires or contraceptive needs, or when contraceptive use is stigmatized and associated with infidelity and/or prostitution [22–25]. Traditional, pro-natalist, religious and cultural practices further exacerbate social norms that discourage the use of contraception in many communities, where the value of having many children is still emphasized in many parts of Uganda today [26, 27].

Qualitative studies that have focused on the role of gender norms in health-seeking behavior often identify unequal and male-dominated power relations between men and women in Uganda’s largely patriarchal society [26, 28]. Previous work focused on men’s attitudes and perceptions towards family planning in Uganda has shown spousal communication, negative beliefs around family planning, fear of side effects such as infertility, and suspicions around infidelity and promiscuity of women who use contraceptives to be factors that influence women’s contraceptive use [29–32]. Traditional gender norms and perceptions dictate that pregnancy, family planning and reproductive health are a woman’s ‘domain’, and thereby exclude men’s involvement [29, 31, 32]. Partner opposition is often a significant predictor of poor healthcare access, unmet need for contraception, the use of traditional rather than modern methods, and clandestine use of contraception [18, 19, 32, 33]. The lack of spousal communication on fertility preference as well as the timing and spacing of pregnancies has often been reported in the literature [18, 26, 30, 31]; coupled with a male-dominant, normative decision-making process, this often results in men making decisions around contraception with little discussion or consultation with their partners [28, 33–35].

In recognising that the motivations and challenges that inform women’s contraceptive use are dynamic and complex, this study seeks to identify the determinants of women’s contraceptive attitudes, intentions and behaviours. This research was part of a larger mixed-methods study examining temporal changes to unmet need for contraception in Uganda [36]. In this paper, we discuss the qualitative findings of focus group discussions (FGDs) on the facilitators and barriers to contraceptive use among women of different ages in both urban and rural settings in the Busoga region of east Uganda.

## 2. Data and methods

### Ethics statement

Ethical approval for the study was granted by the University of Canterbury Human Ethics Committee in August 2017 and by the Mbarara University of Science and Technology Research Ethics Committee in Uganda in October 2017. Participants signed consent forms provided before the FGD (or submitted a thumbprint signature if they were unable to read or write), and were reminded of their right not to answer a question, or to withdraw from the study during the FGD.

### Study setting, design and participant recruitment

The Busoga region was selected due to the low contraceptive prevalence rate (31.3%) and high unmet need (36.5%) among married women of reproductive age [2]. The region is home to approximately four million people, mainly of the Basoga tribe, which is the third largest in Uganda [37]. The region spans about 10,000 square kilometres divided into ten districts [38]. Study sites in the districts of interest (Iganga and Luuka) were selected based on low contraception prevalence rates, accessibility and feasibility.

Six FGDs were conducted with 41 women, stratified across age groups (18–24, 25–34, and ≥ 35 years) and place of residence (urban and rural areas); thematic saturation was achieved after the sixth FGD. Age groups were selected due to cultural norms and literature reporting significant differences in contraceptive uptake across these age brackets [8, 16]. Women of reproductive age (unmarried or married, who were fecund and sexually active; and who wanted to space or limit a pregnancy at the time of the study (irrespective of whether they were using or not using traditional/modern methods of contraception) were eligible to participate. Women who were not sexually active, who were pregnant, or who were trying to conceive at the time of the study were excluded.

Twenty-one women had completed their primary school education, with the other 20 respondents having finished some years of secondary education. Thirty-one women were using some form of contraception at the time of the FGDs (most commonly injectables), while 10 were not using any contraceptive method. Except for 3 women aged 18–24 years, all respondents had at least one child, with 4 respondents ≥ 35 years each having 8 children (Table 1, S1 Data).

**Table 1:** Focus group discussion (FGD) participant characteristics.

Focus group code	No. of participants	Median age years (min, max)	Highest level of education (no.)	Median no. of children (min, max)	Occupation (no.)	Family planning methods (no.)
Rural women (18–24 years)	8	21 (18, 24)	Primary (4), secondary (4)	1 (0, 2)	Student (2), farmer (3), professional (1), peasant (1), businesswoman (1)	Injectables (5), lactational amenorrhea (1), no method (2)
Urban women (18–24 years)	7	23 (18, 24)	Primary (1), secondary (6)	2 (1, 5)	Housewife (4), service worker (1), businesswoman (2)	Injectables (2), implants (1), male condoms (2), female condoms (1), no method (1)
Rural women (25–34 years)	5	27 (25, 29)	Primary (3), secondary (2)	4 (3, 6)	Farmer (4), peasant (1)	Injectables (3), no method (2)
Urban women (25–34 years)	7	27 (25, 32)	Primary (1), secondary (5), tertiary (1)	3 (2, 4)	Professional (2) housewife (4), businesswoman (1)	Injectables (5), oral pills (2)
Rural women (over 35 years)	6	43 (38, 45)	Primary (6)	7 (5, 8)	Farmer (4), businesswoman (2)	Injectables (4), no method (2)
Urban women (over 35 years)	8	40 (35, 38)	Primary (6), secondary (2)	4 (3, 8)	Professional (3), market vendor (2), housewife (2), businesswoman (1)	Injectables (2), oral pills (1), implants (1), male condoms (1) no method (3)

Participants were recruited through purposive sampling, followed by convenience sampling with the help of community health workers and village health teams (VHTs) that worked at these sites. Purposive sampling was initially used to identify participants who met the recruitment criteria at the different study sites, and convenience sampling was used thereafter to continue recruitment till each FGD comprised of 5–8 women. Community health workers recruited participants following a briefing on the nature and topic of the study, the inclusion and exclusion criteria for participants, the ethics of recruitment, the number of participants required, as well as the date, time and location of the FGD in the respective district.

### Data collection and analysis

The FGDs followed the cultural protocols advised by the local partner organizations, for example ensuring refreshments were provided for participants, and that introductions for everyone present were carried out appropriately. Due to the sensitive nature of the topic, protocols regarding informed consent and confidentiality were addressed at recruitment and reiterated prior to the FGD. As participants were recruited by members of health care teams, care was taken to assure women that their participation in the FGD would not affect future care provided by their local family planning clinic or any other health services in their community. As a token of appreciation, participants were given a bar of soap, which was recommended by the local partner organizations as culturally appropriate.

The FGDs were audio-recorded, and usually lasted between 45 minutes to 1.5 hours. They were held in private, quiet spaces where others in the community could not listen in or disturb the discussions. Discussions were guided by the facilitators in Lusoga following a question schedule. Once no new themes were identified in the FGDs, data collection was stopped. Transcripts and subsequent translations of the transcripts to English were checked by two independent translators as well as the facilitator and first author. During the coding and thematic analysis, members of the research team independently checked through and discussed the codes, categories, patterns and themes as they evolved over time.

Qualitative data collected from the FGDs were analysed thematically, using an inductive approach. This method acknowledges how experiences are shaped by context, while retaining an emphasis on how informants make sense of and attribute meaning to their experiences. In analysing the data, the six stages of thematic analysis suggested by Braun and Clarke were followed. The authors analysed the data primarily at a latent level, first using codes to identify patterns within and across the transcripts [39]. Codes were then organised into themes and sub-themes, representing patterns that were identified in the data, incorporating both latent and semantic content. Finally, connections were subsequently built around the major themes that emerged pertaining to the use of contraception by women.

## 3. Results

Using thematic analysis, this study sought a better understanding of why unmet need was high among women in the Busoga region of east Uganda, as well as to determine the facilitators and barriers to contraceptive use among Ugandan women of different ages and living in both urban and rural settings. Three major themes were identified: navigating contraceptive use within a patriarchal context; the influence of community beliefs on women’s contraceptive use; and the importance of women’s own experiences and motivation in contraceptive decisions. These are described below. Where direct quotes are used, U/W refers to urban/rural FGDs, followed by the age group of participants of a particular FGD, and the participant number if applicable; e.g. *U_18–24* indicates a quote from an FGD with urban women aged 18–24 years.

### Navigating contraceptive use within a patriarchal society

The specific gender roles occupied by women and men in a relationship and the dynamics of how those roles play out was identified by participants as an important influence on contraceptive decision-making and uptake. They talked about a woman’s roles of looking after her family and the home, and expectations that they would assume full responsibility of caring for their children. Men, on the other hand, were perceived to want many children but had limited involvement with their upbringing:

*They* [men] *don’t think about it apart from just producing… he doesn’t even take care of educating them*, *as long he produces ‘full stop’*.*—R_25–34*

*Men nowadays don’t think about their responsibilities and you remain with the responsibilities alone so you think of getting many children but for what*! *You just decide to use family planning.—U_25–34*

Contraceptive use was considered to be a woman’s role, but men’s approval was central to women using contraception, as one respondent articulated: “My husband encourages me to go for family planning […] but he almost decides each and everything.” Some younger participants (in the age group 18–24 years) described more egalitarian relationships and shared their partner’s support of contraceptive use. For these women, spousal communication about family size and family planning was more comfortable. For some women, their partner’s instrumental support enabled them to pursue family planning, as described by one participant: “It is my husband who takes me to the health facility for family planning and even if I get problem he takes me back.” This was particularly important in rural settings where participants described long distances and significant transport costs to get to the nearest health facility.

Older women, both in urban and rural areas, were less comfortable or felt unable to discuss contraceptive use with their partners. Partner opposition was framed as a common challenge, despite the burden further births would place on the family. Many older respondents described defying their partners’ wishes, and openly talked about ways in which they subverted partner opposition through covert contraceptive use, mainly through injectable contraceptives. However, the consequences that women face, if discovered by their partners, can be severe and include violence, being abandoned or ‘chased out’ [18, 19, 40]. Many respondents reiterated that attempts at discussing family planning with their partners were unproductive and a source of conflict they sought to avoid:

*For me I just go I don’t tell him (laughs)…now why should you tell him? Because he will ask you many questions…so I think among ten men, only one believes in family planning.—U_≥35*

Partner opposition was attributed by many women to their own negative experiences with contraceptive side effects (e.g. loss of libido, or excessive bleeding) that subsequently interfered with their partners’ enjoyment of sex. In addition to demonstrating typical power dynamics within spousal relationships, these accounts also highlight the expectations that women should please their partners sexually in a relationship:

*Mine does not allow family planning, they will run away from us because of family planning… because some women bleed every day, a month, two months and we don’t have libido so if a man gets another partner, the marriage will break up (laughs)… I am not lying.—U_25–34*

A few younger women cited the costs and hassle associated with side effect management as reasons for their partners being opposed to contraceptive use, as one respondent remarked, “I told my partner after delivering that I wanted to go for family planning until the baby is three years but he refused and told me that if I get any problem I would care for my own self, so I was scared.” The sense of apprehension in some younger women’s comments may be reflective of spousal power dynamics that saw them financially vulnerable in relationships, especially if contraceptive side effects had financial ramifications and men were the primary or sole breadwinner in the family. Older women, on the other hand, described the benefits of family planning in managing the financial burden for their family, even though such concerns were conventionally the purview of the man in a family:

*For me I decided because of the family status… many children but the affordability was less*. *Because even if they send a child from school because of lack of a book*, *he* [the husband] *will just keep quiet… and the child will go to their mother*. *You get your money and give to the child*. *So I decided that if am failing to take care of the six children*, *what if they become eight*, *what will happen*? *So I have to persist on the injectables*.*—R_≥35*

Some women, particularly covert contraceptive users, expressed concern over a lack of confidentiality when interacting with healthcare workers (HCWs), indicating a lack of trust in the provider-client relationship, which then had possible ramifications for their spousal relationships. Low trust with service providers has been reported as an obstacle to seeking out contraceptive services, particularly when providers are part of the same community [25], as one respondent explained:

*… some workers in those clinics don’t keep secrets yet you may not want that information to reach your husband… and as you know woman in spreading gossip, it may reach your husband and you get trouble.—U_25–34*

### The influence of oral testimonials on contraceptive use

Participants discussed some of the societal perceptions of family planning in their communities that either directly or indirectly influenced contraceptive uptake. An example of this was the association of promiscuity and infidelity with women who are contraceptive users, particularly if they were young or unmarried women [see 29, 32, 33, 41]. One participant explained the difficulty such stigma posed for women seeking contraception: “They [young women] face challenges because people talk about them, that what has really forced that young girl to go for family planning! Is it because she is in love with many men?!” Several participants also reiterated that unmarried women who use contraception were perceived to be prostitutes by their community; they explained that the reasoning for this association was driven by the idea that women involved in extra-marital affairs could get pregnant with another man’s child, and therefore used contraceptives to protect themselves.

In discussions about women’s knowledge and opinions about different contraceptive methods, the value and significance of others’ experiences and accounts seemed paramount. Many of the respondents’ comments were preceded by words such as ‘they say’ or ‘people believe’. These accounts were relayed as the beliefs of other women in the community who were either friends, neighbours or relatives of the participants, and was common across women of all ages, and both in urban and rural areas. A few of the testimonies described were positive, and centred on the benefits of family planning. This was particularly common among younger respondents, who highlighted positive aspects such as better health and education of children in a well-spaced family, and an overall better quality of life:

*People have a thought that once they go for family planning, their children will attain better education, because they would have planned for them.—R_18–24*

*People in our community believe in family planning because once you space your children*, *they don’t fall sick so often*, *and even if you have a journey* [travel], *you may not be bothered so much because of the children*.*—R_18–24*

Most participants, however, talked about the harmful consequences of family planning. These centred on negative, sometimes dire health effects heard from other women. Participants’ reliance on other women’s accounts in the community was also apparent from their level of narrative detail around specific family planning methods:

*I was somewhere in Busalamu Health Centre II and women who were using* [the] *injectable method told me that they feel fatigue*, *their legs pain and even their hands cannot lift a hoe and* [they] *feel as if the intestines are burning inside the stomach so I do not know what causes that*.*—R_≥35*

Similarly, some respondents related compelling accounts of other negative experiences that they had heard of, for example:

*I have my sister-in-law, she used family planning when she had given birth to four children but she now has swellings in her abdomen but she has treated those swelling but they don’t heal and she wants to produce girls but she has failed… because of family planning, an injection.—U_25–34*

In many instances, participants spoke of health problems that are not commonly associated with family planning, such as cancer or giving birth to a child with deformities. There was a sense of conviction that contraceptives were to blame for these perceived negative side effects. These accounts were persuasive and convincing that family planning was ‘bad’ because they came from peers, relatives and other women in the community who respondents trusted. However, none of the women seemed to personally know anyone who had experienced these first-hand. A collective, shared sense of fear around possible side effects was embedded in these women’s narratives:

*People in this community think that family planning came to affect people like the use of pills, you may swallow and by the time you want to deliver they check when your uterus has been interfered with and say that the eggs (ovaries) are ‘burnt’.—U_18–24*

Some of the concerns women had were regarding known contraceptive side effects, such as excessive, continuous bleeding or missed menstrual periods, weight changes and a loss of libido. Women who were covert contraceptive users were fearful of these side effects as it was a way for their partners to potentially discover their contraceptive use. A few women, however, were sceptical about the harms and negative beliefs around contraception. It was evident they based these opinions on their own experiences and the benefits they had derived from family planning:

*Some people say that once you go for family planning you don’t produce, you become barren… so they discourage people but I think they are lying because I have used it when I was still at school but when I dropped it, I gave birth immediately.—U_25–34*

### Women’s contraceptive choices are guided by experience, hardship and motivation

Women who were contraceptive users mentioned a few pivotal considerations in their choice of a contraceptive method. Convenience was a critical factor, particularly when it came to deciding between a short- or long-term method. The hassle of adhering to a daily routine with some short-term methods was emphasized, particularly if this had to be done surreptitiously:

*The disadvantages with some methods of family planning is that you get inconvenienced especially when you don’t count the days so well and yet at the same time you have kept it as a secret to your husband. Another one is pills which you may easily forget and miss a day or so.—R_18–24*

Respondents who had used long-term methods brought up the inconvenience and costs of having to revisit a trained professional for their implant (or more rarely, intra-uterine device [IUD]) to be removed. Many had since switched to a short-term method:

*For me I use Injectaplan but I don’t have any problem* […] *You don’t suffer to go there*, *to look for the doctor*, *as it happens with IUD*. *With IUD you have to deal with the doctor who inserted it and when you find him*, *they don’t remove it*. *An injection is easier to drop*.*—R_25–34*

*The reason I prefer an injection is because an IUD takes three years and if you want to remove it before it expires, they tell you to pay 20,000 to remove it… and pills would work better but you may forget to swallow them and even the IUD we fear that it has side effects, making us just left to use injections.—U_25–34*

Women’s familiarity and satisfaction with a method was also key in deciding which method to use, as one respondent said, “For me I decide once, say on pills or injectables, because it’s [what] I want… Because I am used to that and I have enough experience in it and it’s what I know and can manage.” When asked if they were satisfied with their current method of choice, participants responses were mixed; some women continued to use a method because the perceived benefits outweighed the challenges they faced, but other women switched or discontinued methods due to side effects. Some participants shared their own personal accounts of the negative side effects of contraceptive use they had experienced. These were mostly related to menstrual irregularities, as well as health effects and pain that interfered with their daily activities, particularly when manual labour was involved:

*Family planning makes us women weak, we feel dizzy, back pains and you can’t walk for a long distance because you feel fatigue.—U_≥35*

The management of these side effects became problematic when participants had to make repeat visits to the clinic, or bled for very long periods of time; however, on the whole they still benefited from contraceptive use, and therefore persisted despite the challenges they faced:

*For me I am not happy because I don’t go into my periods as usual but I may have scanty blood, then after two days, I bleed again and I feel headache and pain in the legs so I have problems with [it] but it has helped me not getting pregnant.—U_18–24*

The use of family planning specifically for limiting births was not mentioned by many participants; instead, most women talked about contraceptive use from the perspective of spacing and permanent methods were rarely discussed. When participants talked about long-term methods, they were not discussed in the context of limiting children, but rather in terms of longer spacing between pregnancies.

A few participants, particularly in rural areas, mentioned some of the traditional family planning methods that they had heard of being used ‘in the villages’. Though these accounts had come from other women in the community, the way in which participants described them seemed to imply a sense of certainty in their effectiveness:

*There are herbs which you mix with the first menstrual blood, you tie it together, then you hang it up at the front of the door, and once you want to get pregnant, you just remove it and dip it in water.—R_18–24*

Some respondents also talked about emergency contraception and abortions as contraceptive options in situations involving unprotected sex and unplanned pregnancies, respectively. However, none of the respondents had personal experience with using these methods themselves; instead, they again described what they had heard from *other* women. Knowledge around emergency contraception was minimal and in some instances, non-existent. Only a few participants had heard of ‘medicines’ that health workers could give a woman to avoid a pregnancy. The respondents’ reliance on other women’s accounts or experiences as a source of information came through strongly, and most participants talked about home remedies they had heard of or been told about:

*I hear that if you have unprotected sex with a man, immediately after sex, squat and squeeze yourself until those things (sperm) get out and a chance may come that you don’t get pregnant.—U_25–34*

It is worth noting that these methods were predominantly mentioned by younger participants. Older participants had no information about avoiding a pregnancy after unprotected sex; most of them stated they had no options in such situations.

Where participants supported abortion as an option, often this was restricted to a situation where a woman’s circumstances were considered to justify terminating a pregnancy, such as illness or an extramarital pregnancy. Others considered abortion itself to be unsafe, as one respondent shared: “If I get an unplanned pregnancy, I just wait until I give birth. Because aborting has two options, either to survive or die, so I just deliver”.

## 4. Discussion

This qualitative study sought to explore the contextual factors and barriers that determine women’s contraceptive decisions and uptake in the Busoga region of east Uganda. The themes identified in this study highlight how socio-cultural and gendered contexts influence women’s use of contraception. While shouldering the bulk of the contraceptive burden, many women recognised and valued contraceptive use for the benefits they had experienced; others highlighted the persistent challenges to access and use of contraception. These included norms that dictated a woman’s ability to make choices regarding her reproductive health; partner opposition to contraceptive use; and fears about the harms of contraceptives. Despite these fears and challenges, several respondents also acknowledged the personal benefits of contraceptive use, and many women resolutely sought out contraceptives in order to manage the size of their family and handle the responsibilities and roles they had as women in their families and societies, which aligns with previous findings in the literature [42]. This was sometimes done at the risk of severe consequences on the part of their partners, particularly if they were discovered using contraception covertly.

Although there has been progress in the areas of gender equality and equity in several African countries over the last few decades, many countries, including Uganda, are still represented by largely patriarchal, patrilineal, male-dominant societies, norms and practices [30]. Such norms discourage or impede spousal communication about family size and spacing between pregnancies, and enforce the expectation that women must respect and conform to the decisions made by their partners [25, 30, 33]. Women’s narratives in this study reiterated the complex interconnectedness of gender roles, power dynamics, male-dominance and the expectations of women and men in a relationship, and how these can influence women’s decisions and actions around contraceptive use. Few participants however, linked partner opposition to contraceptive use solely as a way for their partner to assert their power and authority. Rather, partner opposition was attributed to the loss of sexual pleasure, concerns about family planning methods and side effects, and the financial inconveniences of side effect management.

Discordant fertility desires between men and women regarding the number of children and family size have been reported previously in the literature, with men usually wanting more children than their partners [43]. Coupled with a lack of spousal communication and men’s expected and accepted role as primary decision maker, women are often unable to negotiate contraceptive use [40]. These women’s circumstances strongly motivated them to exert their own agency in seeking out contraceptive services to space or limit subsequent pregnancies. While spousal communication and joint decision-making has been associated with increased contraceptive use in many studies [30, 34, 44], few interventions have shown success in achieving sustained changes to couples’ communication over time. In the absence of such discussions, covert contraceptive use has long been acknowledged as a way for women to exert control over their own fertility, manage the size of their family and look out for the welfare of their children [25, 45–47]. For these respondents, covert use seemed to be an ordinary and accepted practice, an ‘open’ secret among most women and health care providers, held together by a shared understanding that this was the only option available to many women. Participants understood the serious consequences they would face if their contraceptive use were discovered by their partners, yet the benefits of spacing or limiting births outweighed the risks for them.

Very few studies have looked at how gender norms in Uganda have evolved over time, and how these could influence younger people’s attitudes and contraceptive behaviour. The findings from younger FGDs may be reflective of recent observations that younger people want small, well-spaced families, which sometimes has meant challenging existing gender norms and societal expectations in order to achieve this [25, 48]. Results of more recent initiatives and programs around family planning have also shown that younger Ugandans, particularly educated men, have different attitudes and opinions about family sizes, and are more open to the use of family planning for spacing and limiting of pregnancies [28, 35]. That younger women felt more comfortable and open with their male partners in reproductive health discussions may be an indirect result of this change in mind set among younger Ugandan men, together with an increased openness and awareness around reproductive health. An interim review of Uganda’s FP-CIP 2015–2020 targets, for instance, mentions that demand creation for family planning among men and younger Ugandans has been successful, with peer educators being engaged and supported to educate these groups about family planning [49].

Where contraceptive methods were concerned, the convenience of long-term methods detailed by some respondents included less frequent visits to the clinics and less cumulative costs incurred for repeat contraceptive prescriptions. Yet many women mentioned a lack of trained service providers for these methods, and therefore faced challenges in accessing these options readily, as well as seeking out providers who could facilitate the removal of these methods when women wished to discontinue them. Though there has been a recent push for the distribution and provision of long-term methods at the national level [50, 51] and a directive from the Uganda FP-CIP for increasing the number of providers trained to administer these, the realities on the ground for many women are restricted by the factors mentioned above. At the health system level, ensuring the continued procurement and availability of stocks of contraceptive methods [52], as well as increased resources and training for HCWs in the administration of long-term methods, could go a long way in meeting women’s demand for contraception to space or limit their pregnancies.

In Uganda’s largely collectivist society, the strength of ties and relationships within one’s community are critical to continuing cultural norms and socially acceptable behaviours, including behaviours related to health [53, 54]. In this study, the majority of participants were fearful of negative contraceptive effects, both real and perceived, even if these were based only on stories they heard from other women. The association of contraceptive use with threatening health outcomes such as cancer, infertility and congenital deformities was common, and instilled fear among potential contraceptive users and the community, as has been reported in previous studies [17–20]. These findings may signify the limited success of educational efforts at health facilities and community-level discussions led by VHTs and mobile outreach clinics in addressing these concerns around family planning [42, 55–57]. However, as evidenced from the testimonies in this study, women of all ages and in both urban and rural settings held the opinions and experiences of other women in the community in high regard, and knowledge shared among community members appear to continue perpetuating these beliefs. The power of these testimonies override information about family planning from other sources due to a sense of trust among peers, particularly within the informal networks among women, as has been reported previously [21, 58, 59].

The power of testimonial information and community beliefs was integral in shaping the attitudes of women towards contraceptive use, as well as being a source of caution and fear. This was particularly significant when it came to the opinions of extended family members as well as religious beliefs, and this in turn impacted women’s decision-making and use of contraception. Village elders, older family members and in-laws, in particular, are known to have great influence over newly married couples and fertility decisions [26]. Socio-cultural norms appeared to differ in their effect across the life course among women; for example, younger women talked more about societal pressure to have a child soon after marriage, while older women spoke about how the practice of polygyny influenced their contraceptive behaviour. Together with norms that allow polygynous relationships, discourage premarital sex, dictate expectations around family size and how soon after a marriage a child is expected, these multi-level factors collectively influenced a woman’s contraceptive behaviour and decisions. The reliance on FGDs was a strength of this study in offering rich, personal data around attitudes, knowledge and experiences with contraception. First-hand accounts and experiences about contraceptive use were provided by women from the Busoga region, which was the geographical area of interest in this project due to its low contraceptive prevalence and high unmet need. Therefore, the data gathered may have exemplified specific ethnic, tribal or geographical influences on contraceptive use, though these factors were not specifically questioned or raised in the FGDs. The use of FGDs were useful for elucidating the social attitudes and collective, shared knowledge about contraception, as well as understanding the importance of informal networks among women when it came to exchanging information and seeking reassurance about reproductive health matters and contraceptive decisions.

Nonetheless, the study is not without its limitations. As all FGDs were conducted in Lusoga, some of the nuances, jargon and cultural ways of describing or thinking about certain topics or issues may have been lost in the process of transcription and translation by the local Ugandan research team. The presence of staff from the local partner organizations and the first author being a foreign researcher may also have influenced responses in terms of honesty and participant bias. Relatedly, the analysis of certain cultural references or practices may have lacked the specific contextual understanding that a Ugandan researcher might have had. However, in order to mitigate this, all local and cultural references in the transcripts were cross-checked and discussed with the Ugandan research team. A limitation of the thematic analysis approach was that linguistic nuances in the ways respondents described or talked about certain topics were not given due attention; a discourse or narrative analysis may have better captured these elements of the data.

A number of areas related to the topics covered in this study remain unexplored and in warrant of further investigation. Though some research exists on how decisions are made about contraceptive use among couples, there is less understanding about the nuances of couple communication (or a lack thereof) regarding family size, fertility preferences and contraceptive decision-making, particularly from studies that obtain accounts from both members of a couple. Insights on the negotiation processes that women engage in would be valuable in expanding our understanding of how cultural norms, gender dynamics, relationship inequalities and women’s status affect contraceptive decisions. Equally unknown are the mechanisms of contraceptive decision-making among women and men who are unmarried but sexually active, and those in polygamous relationships.

Informal networks and community knowledge were highlighted as a key source of information and influence on contraceptive decisions and uptake. While previous studies have highlighted how rumours and ‘hearsay’ in communities can affect contraceptive behaviour, little research exists on how these informal networks are created, how information is communicated in the community. Findings from such a study could inform community-level interventions aimed at dispelling fears about contraceptive use, disassociating female contraceptive use from infidelity and/or prostitution and perhaps change the narratives that are passed on through informal networks in communities.

## 5. Conclusion

This study sought to explore the key determinants of contraceptive decision-making and uptake in Uganda. The findings presented are important for informing health policy and program direction, particularly for addressing unmet need for contraception among women. A consideration of the barriers still faced by women in seeking out and using contraception allows for a more nuanced design of interventions at the individual, familial and societal levels. Partner opposition to contraception and low male involvement remain definitive challenges to be addressed, more so in the patriarchal context of Ugandan society, where socio-cultural and gender norms operate largely in favour of men’s dominance and women’s subordination. While cultural norms in Uganda do not overtly promote discussions about fertility, family size and child spacing among couples, this study highlights changing mind sets and attitudes that allow for more open communication about such topics, particularly among younger Ugandans. Family planning and integrated health programs should continue to facilitate communication and shared decision-making among couples, while recognising the gender dynamics that dictate women’s and men’s role in relationships. And while it may be unrealistic to expect rapid shifts in ways of thinking about gender norms and societal expectations of women and men in Uganda, it remains critical that initiatives advocating for a more egalitarian and less patriarchal perspective around gender equality and women’s status continue.

## Supporting information

S1 Data FGD participant data. (XLSX)

S1 Text Inclusivity in global research. (DOCX)
